# Mosquito Consumption by Insectivorous Bats: Does Size Matter? 

**DOI:** 10.1371/journal.pone.0077183

**Published:** 2013-10-10

**Authors:** Leroy Gonsalves, Brian Bicknell, Brad Law, Cameron Webb, Vaughan Monamy

**Affiliations:** 1 School of Arts & Sciences, Australian Catholic University, North Sydney, New South Wales, Australia; 2 Forest Science Centre, Department of Primary Industries, North Parramatta, NSW, Australia; 3 Department of Medical Entomology, Pathology West, Institute for Clinical Pathology and Medical Research, Westmead Hospital, Westmead, NSW, Australia; 4 Marie Bashir Institute for Infectious Diseases and Biosecurity, University of Sydney, Westmead, NSW, Australia; University of Regina, Canada

## Abstract

Insectivorous bats have often been touted as biological control for mosquito populations. However, mosquitoes generally represent only a small proportion of bat diet. Given the small size of mosquitoes, restrictions imposed on prey detectability by low frequency echolocation, and variable field metabolic rates (FMR), mosquitoes may not be available to or profitable for all bats. This study investigated whether consumption of mosquitoes was influenced by bat size, which is negatively correlated with echolocation frequency but positively correlated with bat FMR. To assess this, we investigated diets of five eastern Australian bat species (*Vespadelus vulturnus* Thomas, *V. pumilus* Gray, *Miniopterus australis* Tomes, *Nyctophilus gouldi* Tomes and *Chalinolobus gouldii* Gray) ranging in size from 4-14 g in coastal forest, using molecular analysis of fecal DNA. Abundances of potential mosquito and non-mosquito prey were concurrently measured to provide data on relative prey abundance. *Aedes vigilax* was locally the most abundant mosquito species, while Lepidoptera the most abundant insect order. A diverse range of prey was detected in bat feces, although members of Lepidoptera dominated, reflecting relative abundance at trap sites. Consumption of mosquitoes was restricted to *V. vulturnus* and *V. pumilus*, two smaller sized bats (4 and 4.5 g). Although mosquitoes were not commonly detected in feces of *V. pumilus*, they were present in feces of 55 % of *V. vulturnus* individuals. To meet nightly FMR requirements, *Vespadelus* spp. would need to consume ~600-660 mosquitoes on a mosquito-only diet, or ~160-180 similar sized moths on a moth-only diet. Lower relative profitability of mosquitoes may provide an explanation for the low level of mosquito consumption among these bats and the absence of mosquitoes in feces of larger bats. Smaller sized bats, especially *V. vulturnus*, are likely to be those most sensitive to reductions in mosquito abundance and should be monitored during mosquito control activities.

## Introduction

Mosquitoes may cause serious nuisance biting and serve as vectors of mosquito-borne pathogens such as Ross River virus (RRV) and Barmah Forest virus (BFV) [1]. In response to the risk posed to public health by mosquitoes, broadscale mosquito control programs have been implemented around the world to mitigate the risk of irruptions in the number of cases of mosquito-borne arbovirus infections and nuisance biting [2,3]. In some instances, reductions up to 98 % in larval populations have been achieved [4].

Insectivorous bats are often touted as a potential biological control for mosquito populations. Many of these claims stem from the study of Tuttle [5] that suggested that bats may serve as an alternative approach to broad-scale mosquito control, with a single bat capable of consuming up to 600 mosquitoes per hour. More recently, Reiskind and Wund [6] also suggested a possible role for bats in the reduction of disease vectors after observing a 32 % reduction in oviposition by *Culex* spp. associated with bat predation. However, the suggestion by Tuttle [5] was based on an extrapolation from the laboratory study of Griffin et al. [7], that like the study of Reiskind and Wund [6], did not account for a range of other factors such as satiation of bats, the abundance of mosquitoes relative to other prey, the ability of bats to detect mosquitoes amongst various levels of vegetative clutter, the digestibility of mosquitoes as well as the calorific requirements of bats, all of which will presumably influence the degree to which bats consume mosquitoes.

Though bats of various sizes (small to large) have been observed consuming mosquitoes [7,8] and mosquitoes have been identified in the stomach contents of bats [9,10] as well as in bat feces [8,11,12], many dietary studies have found mosquitoes to represent only a small proportion of bat diet, with other groups (e.g., moths and beetles) more common in bat diets [13]. However, the importance of mosquitoes and other small, soft-bodied prey may be understated due to the greater susceptibility of soft-bodied prey to the processes involved in mastication and digestion [14,15] and the bias associated with techniques available to study bat diet [14,16] . Recent advances in molecular techniques have allowed greater resolution of animal diets, particularly for cryptic animals that are difficult to observe foraging. Since these techniques rely on DNA for the identification of prey, either in gut contents or in feces, detection of soft-bodied prey may be improved. 

In systems where mosquitoes are highly abundant, particularly during summer months [17], mosquitoes potentially represent a small sized (~5 mm) prey resource for insectivorous bats. In the study area, activity of only small bats of the *Vespadelus* genus (*V. vulturnus* and *V. pumilus*) was positively correlated with the abundance of *Ae*. *vigilax* [17]. Additionally, *V. vulturnus* shifted foraging ranges relative to changes in the distribution and abundance of *Ae*. *vigilax* [18], suggesting that the mosquito may be an important prey item in the study area. However, given the small size of mosquitoes, they may not be available to all insectivorous bats. Echolocation call structure that influences the ability of bats to forage within habitats of varying clutter [19,20,21] is also thought to influence the size of prey that bats are able to successfully locate [22]. Since larger bats tend to use low-frequency echolocation to detect prey, the longer wavelength associated with this echolocation is considered to be unsuitable for detecting small prey such as mosquitoes [22]. However, the diet of a number of medium to large sized European bats does not support this theory in that small prey were frequently consumed [23,24,25].

The energetic requirements of bats may also serve to diminish the use of mosquitoes as prey by bats. Since field metabolic rate (FMR) increases as a function of mass [26], larger bats are required to obtain more energy each night than smaller bats. Given, the lower calorific value of mosquitoes relative to other insect taxa [27,28], larger bats may be not be able to meet FMR requirements by eating mosquitoes alone. 

To investigate the influence of bat size on consumption of mosquitoes, five species representing a range of sizes (small – large) were sampled and their diets investigated using molecular techniques to identify which species consume mosquitoes and potentially are more vulnerable to reductions in prey abundance resulting from broadscale mosquito control. Four of the five bat species studied employ relatively high-frequency echolocation (>50 kHz) thought to be more suited for detection of small prey [22]. We concurrently measured the abundance of mosquitoes and other insects through the course of one field season to provide data on the availability of prey and predicted that consumption of *Ae*. *vigilax* would be restricted to smaller bats that are influenced by the abundance of mosquitoes [17,18].

## Methods

### Ethics statement

Since all trapping locations were on NSW National Parks and Wildlife Service estate, all work (harp trapping, light trapping and EVS trapping) was carried out under scientific licence (S12771) issued by the NSW National Parks and Wildlife Service. 

Animal ethics permits (TRIM no. TRIM 09/6902 (6)) for harp trapping (for the purposes of collecting bat fecal matter) were obtained from the NSW Director-General's

Animal Care and Ethics Committee (DG’s ACEC). Harp trapping of a threatened species was undertaken as per conditions of the scientific licence and animal ethics permits (records of bats trapped and injuries to be reported to animal ethics committee at conclusion of field work). 

### Study site

**Figure 1:**
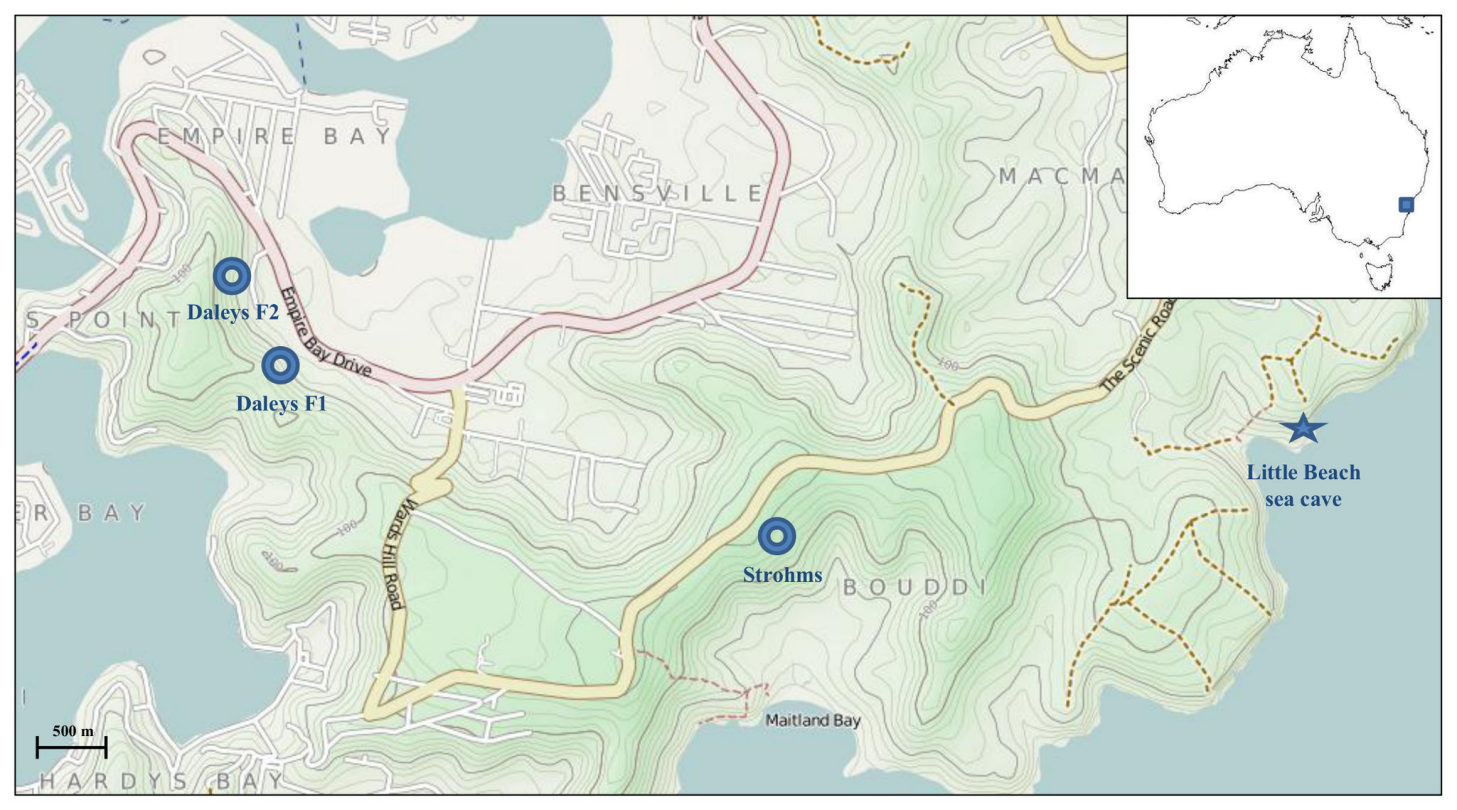
Sampling sites within study area (inset: map of Australia indicating relative location of study area). Maps are adapted from © OpenStreetMap contributors (http://www.openstreetmap.org/copyright). Donuts represent harp trapping locations along Daleys Point and Strohms fire trails in Bouddi National Park. Star represents location of sea cave in which *Miniopterus australis* individuals were trapped in 2011.

The study area was located in the Empire Bay region (33°29’57”S, 151°21’40”E) of the Central Coast of New South Wales, Australia (Figure 1). This region is approximately 50 km north of Sydney and experiences a warm sub-tropical climate. Within the study area, a large national park (Bouddi National Park) sustains populations of hollow and cave roosting insectivorous bats, including six threatened species listed under the NSW *Threatened Species Conservation Act 1995* [29]. Large estuarine areas (primarily coastal saltmarsh and mangrove forests) that represent important larval habitats for *Ae*. *vigilax* throughout the austral summer are also present [17]. Sampling was undertaken 1-2 km from estuarine habitats in Narrabeen Coastal Blackbutt Forest where adult *Ae*. *vigilax* is abundant and bats are most active in the study area [17]. This vegetation community has a typical canopy height of 20 m, occurring on Narrabeen sandstone that supports a sparse-to-moderate understorey of shrubs and a well developed grass layer [30]. Canopy cover in this vegetation community is approximately 40 %. 

### Collection of bat feces

Bat trapping was conducted using harp-traps [31] set at three sites along flyways on two fire trails within Bouddi National Park. Traps were not set in saltmarsh habitats where *Ae*. *vigilax* emerges because of the difficulty of trapping bats in open habitats and since bats are less active in this habitat [17]. Each trap was checked and cleared at midnight, as well as at first light. Captured bats were removed from traps, placed into individual calico holding bags (autoclaved) and processed (including identification and collection of morphometric data). All individuals were held for one hour to defecate if trapped during the first half of the night, or until dusk the following night, at which time they were released at the point-of-capture. In February 2011, harp traps were set in a sea cave used by the little bent wing bat (*Miniopterus australis*) Captured bats were immediately removed from traps, placed into calico bags and transported out of the cave to be processed (as above). Individuals were held overnight to defecate before release from the point-of-capture. Feces produced by bats in calico bags were transferred to 1.5 mL microcentrifuge tubes and were frozen immediately to reduce the degradation of fecal DNA. 

The selection of bat species for analysis was based on predicted minimum detectable prey size, mass and trapping frequency (Table 1). The species selected for analysis were Gould’s wattled bat (*Chalinolobus gouldii*), little bent-wing bat (*M. australis*), Gould’s long-eared bat (*Nyctophilus gouldi*), eastern forest bat (*Vespadelus pumilus*) and little forest bat (*V. vulturnus*). The five species range in mass from 4-14 g and with the exception of *C. gouldii* (25-34 kHz), all bats employ frequency modulated echolocation calls with terminal frequencies >50 kHz. Additionally, *N. gouldi* is often considered to be a gleaning bat because of the steep linear nature of its calls and its use of passive listening as a hunting strategy (see[32]). The terminal portion of its calls is often < 50 kHz (see 33). 

**Table 1:** Minimum detectable prey size and energetic requirements of each bat taxa recorded in this study.

**Species**	**No. trapped individuals**	**Mass (g)**	**Echolocation frequency (kHz)**	**Min detectable size^a^ (mm)**	**Detectability of mosquito (5.2mm)**	**FMR^b^(kjd^-1^)**	**No. mosquitoes required^c^**	**No. moths required^c^**	**Foraging time^d^ (hrs)**	
									**Mosquito**	**Moth**
*C. gouldii*	5	13.8	34	8.1		44.31	2139	582	10.2	4.8
*Miniopterus australis*	7	6.7	65	4.2	✓	26.11	1260	343	6.0	2.9
*Nyctophilus gouldi*	10	12.3	80	3.4	✓	40.73	1966	535	9.4	4.5
*V. pumilus*	10	4.4	53	5.2	✓	19.51	942	256	4.5	2.1
*V. vulturnus*	20	4	53	5.2	✓	17.89	863	235	4.1	2.0

a Predicted minimum detectable prey size using equation of Møhl [52].

b Field metabolic rate using equation of Speakman and Thomas [26].

c Number of prey required to meet FMR assuming energy absorption of 70 % [49]; calorific value of mosquito (0.002 g) = 14.8 kJg^- 1^ [27] , moth (0.004 g) = 27.2 kJg^- 1^ [28]

d Foraging time required to obtain enough mosquitoes or moths (5–10 mm) to meet FMR requirements assuming attack rate of 5 min^-1^ and 70 % success for mosquitoes and 40 % success for moths [53].

### Collection of prey abundance data

The population abundance of *Ae. vigilax* is driven heavily by tidal and rainfall inundation of larval habitats (i.e., coastal saltmarsh and mangrove communities). General patterns such as peaks in population abundances can be predicted [34], with larger populations tending to be present two weeks after inundation of saltmarshes by spring tides and/or heavy rainfall. To encompass the variation in *Ae*. *vigilax* population abundances, sampling was undertaken over two consecutive nights in each of eight fortnights during spring and neap tides during the austral summer of 2009/10, at times that coincided with either predicted large or predicted smaller mosquito population abundances. The mosquito fauna at each site was surveyed concurrently with bat trapping using one CO_2_-baited encephalitis virus surveillance (EVS) trap [35] (Australian Entomological Supplies, Bangalow, NSW, Australia). Other aerial insect fauna were sampled concurrently with mosquito sampling using one standard light trap (Australian Entomological Supplies, Bangalow, NSW, Australia). All specimens were killed by being placed into dry-ice, stored dry and frozen. Mosquito collections were identified to species according to keys [36] and the nightly abundance of each species was recorded. Light trap collections were sorted into three Orders (Lepidoptera, Coleoptera and Diptera), with all other specimens pooled into an ‘other’ category. The nightly abundance of each insect order was recorded.

### Analysis of bat feces

Genomic DNA was extracted from a pooled sample of five fecal pellets for each trapped individual using a commercial DNA extraction kit (Methods S1). A 157bp section of the DNA barcoding region, cytochrome oxidase I was amplified using taxon-specific primers, ZBJ-ArtF1c and ZBJ-ArtR2c [37], purified and cloned. DNA from a sub-sample of 16 clones from each clone library was then sequenced at the Australian Genome Research Facility (Westmead Millennium Institute, Sydney) (Methods S1). DNA sequences were trimmed of flanking vector and entered into the identification engine on the barcoding of life database (BOLD). The nearest sequence match and percent similarity of each sequence was recorded, with a taxonomic assignment to order, family, genus or species using taxonomic assignment thresholds [36]. Sequences with low similarity (<92 %) to reference sequences in BOLD were excluded. See Results S1 for DNA sequences.

### Calibration of technique sensitivity for detection of mosquito DNA

While Zeale et al. [37] reported that taxon-specific primers were able to detect a wide variety of taxa, mosquitoes were not included in testing of the primers. To provide a baseline for detectability of mosquito DNA amongst DNA of other taxa, artificial bat feces was manufactured with two prey items: mealworms (*Tenebrio molitor*, L.) and mosquitoes (*Aedes aegypti*, L.). Artificial bat feces consisting of 0%, 5 %, 10 %, 15 %, 20 % and 100 % of mosquito (by volume - representing 0, ~6, ~11, ~17, ~22, ~110 mosquitoes, respectively) were made by adding an appropriate volume of mosquito slurry for each concentration and then adding a volume of mealworm mixture that accounted for the lower concentration of mosquito material relative mealworm material in the stock solutions (0.18 g mL^-1^ and 0.30 g mL^-1^) (Supporting Information – Methods S1). Each solution was vortexed vigorously for 1 min to mix the mosquito and mealworm material. Once mixed, 1 mL of the mixture was used to extract DNA for use in PCR as described above for bat feces, except there was no cloning step. Each mosquito concentration was treated in triplicate. If after sequencing, a sequence appeared to be mixed, it was inferred that both the mosquito and mealworm DNA had been amplified. To confirm this, one PCR product from each mosquito concentration was cloned and ten clones were sequenced from each clone library. 

### Data analysis

Repeated measures-analysis of variance (RM-ANOVA) was used to test the significance of differences in mean nightly *Ae. vigilax* population abundances between spring and neap tides. Additionally, RM-ANOVA was used to test the significance of differences in mean nightly abundance of all insects and each insect order between spring and neap tides. For each bat species, the relative importance of each insect order to bat diet was based on the frequency of occurrence of the insect order (i.e., percentage of sample size that contained a DNA sequence matching a taxon within that order). A chi-square goodness of fit test was used to investigate whether consumption of each prey taxa reflected prey availability. For each bat species (except *M. australis* for which no prey abundance data were collected), the percentage of identified prey items belonging to each insect order was compared with the percentage of each insect order represented in light trap collections. Pearson correlation analysis related prey abundance and detection of *Ae*. *vigilax* in bat feces.

## Results

### Prey abundance

A total of 12 898 mosquitoes was collected during the study representing 14 mosquito species (Table 2). Of these, 12 were recorded during spring and neap tides, respectively. The most abundant species irrespective of tidal cycle was *Ae*. *vigilax*, representing 77.81 % of all mosquito species recorded during spring tides, and 83.9 % of all mosquito species sampled during neap tides. The other commonly collected species were *Ae. multiplex* (Theobald), *Ae*. *notoscriptus* (Skuse) and *Culex sitiens* (Wiedemann).

**Table 2:** Nightly abundances (averaged across sites) ± standard error of mosquito species trapped during spring and neap tides.

**Species**	**Spring**	**Neap**
*Aedes alternans*	3.40 ± 0.53 (1.15)	7.12 ± 1.24 (1.64)
*Aedes multiplex*	15.12 ± 3.12 (5.14)	11.19 ± 4.72 (2.58)
*Aedes notoscriptus*	12.77 ± 3.50 (4.34)	8.24 ± 0.68 (1.90)
*Aedes procax*	4.10 ± 0.87 (1.39)	5.27 ± 0.87 (1.21)
*Aedes rubrithorax*	0.10 ± 0.10 (0.03)	
*Aedes vigilax*	229.10 ± 60.00 (77.81)	364.67 ± 86.96 (83.93)
*Anopheles annulipes*		0.05 ± 0.05 (0.01)
*Coquillettidia linealis*	0.05 ± 0.05 (0.02)	0.05 ± 0.05 (0.01)
*Culex annulirostris*	12.20 ± 5.8 (4.14)	15.31 ± 4.99 (3.52)
*Culex australicus*		0.1 ± 0.07 (0.02)
*Culex molestus*	4.10 ± 0.77 (1.39)	2.13 ± 0.11 (0.49)
*Culex qinquefasciatus*	2.71 ± 0.47 (0.92)	2.15 ± 0.18 (0.49)
*Culex sitiens*	10.72 ± 4.12 (3.64)	18.23 ± 7.43(4.20)
*Tripteroides atripes*	0.05 ± 0.05 (0.02)	
Total	294.42 ± 99.97	434.80 ± 100.00

NB. Values in brackets represent percent of total mosquito abundance in each habitat.

**Figure 2:**
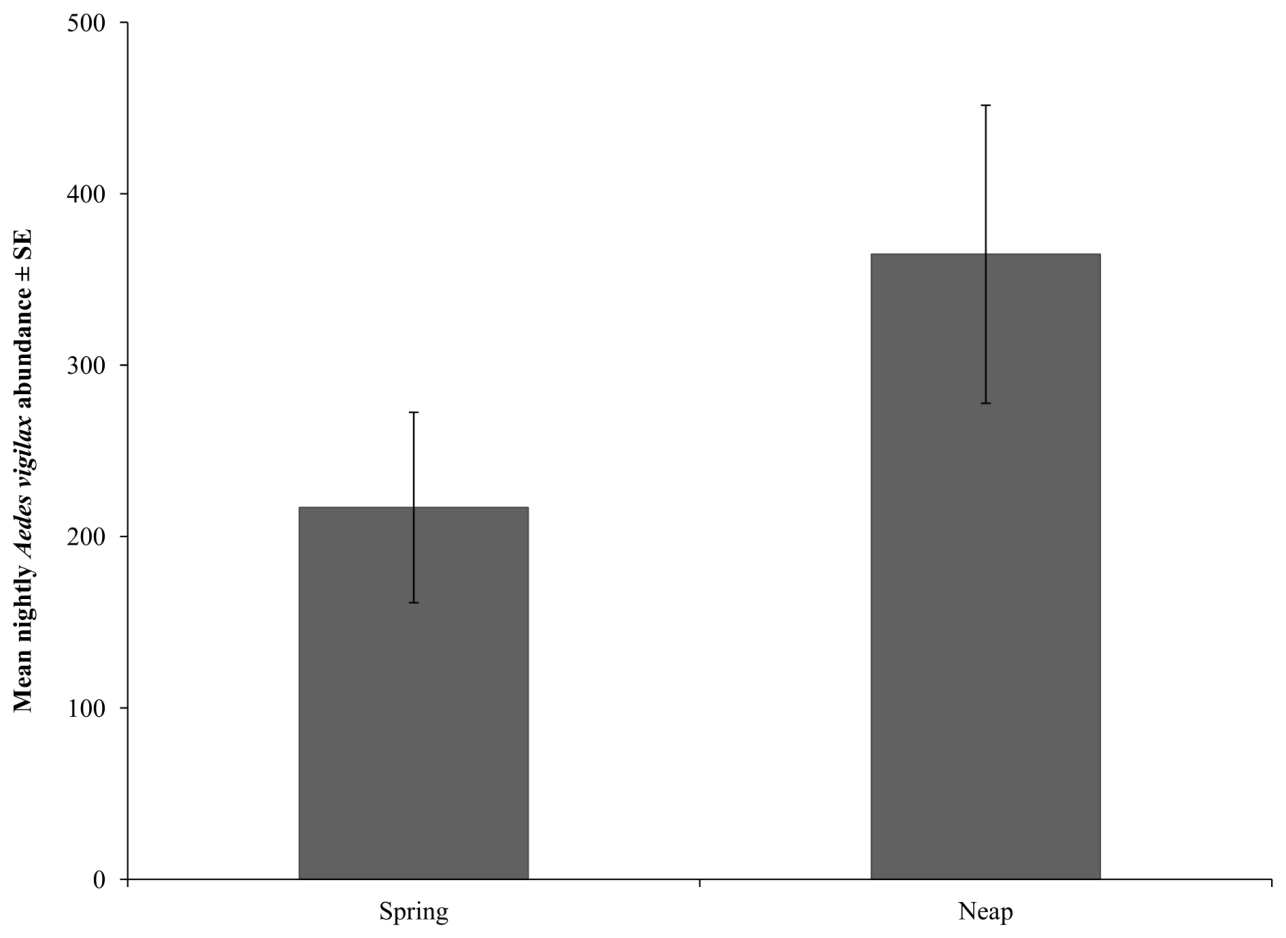
Nightly *Aedes vigilax* abundance. *Aedes vigilax* abundance during spring and neap tides.

As predicted, nightly *Ae. vigilax* abundance was greater during neap tides (364.67 ± 86.96), however it was not significantly different from spring tides (229.10 ± 60.00) (F(1) = 2.125. p = 0.152; Figure 2). 

**Figure 3:**
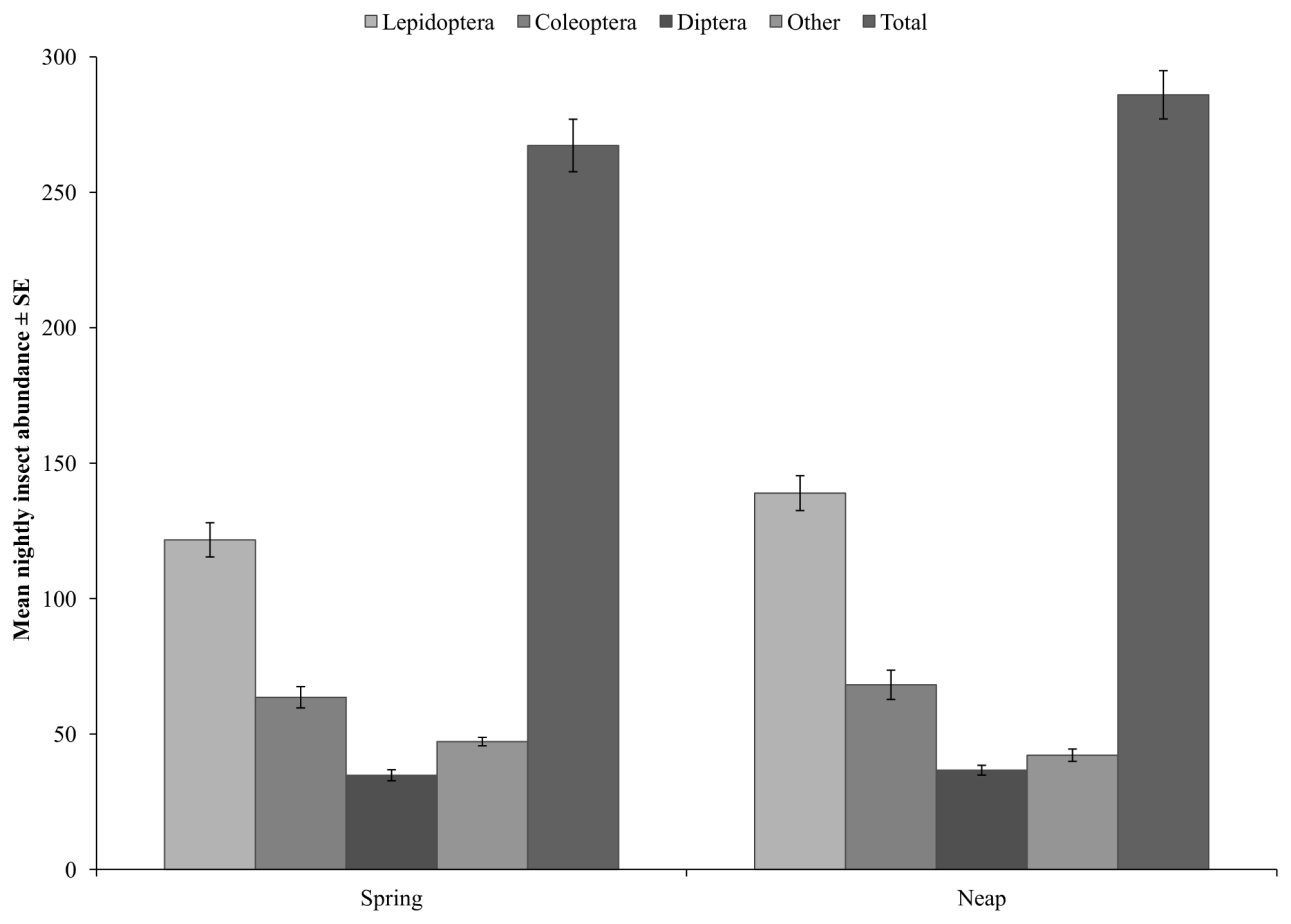
Nightly insect abundance. Insect abundance during spring and neap tides.

Total nightly insect abundance during spring tides (267.29 ± 9.69) was not significantly different to total nightly insect abundance during neap tides (286.00 ± 8.90) (F(1) = 1.982, p = 0.166; Figure 3). Lepidopterans, Coleopterans, Dipterans and ‘other’ taxa, consisting of representatives of Blattodea, Hemiptera, Hymenoptera, *Isoptera*, Odonata and Orthoptera, were recorded in light trap collections. Irrespective of tide height, Lepidopterans were the most abundant taxa in light trap collections, representing 45.5% and 48.6% of all insects trapped during spring and neap tides, respectively. Coleopterans were the second most abundant taxa, representing 23.8% of all insects trapped during both spring and neap tides, while dipterans were less abundant, representing 13% and 12.8% of all insects during spring and neap tides, respectively. All other taxa represented 17.7% and 14.8% of insect collections during spring and neap tides, respectively. The abundance of Lepidopterans, Coleopterans, Dipterans and all other taxa did not differ between spring and neap tides (F(1)=3.632, p=0.063; F[62]=0.491, p=0.487; F[62]=0.462, p=0.500; F[62]=3.463, p=0.070; Figure 3). 

### Bat diet

#### Calibration of technique sensitivity for detection of mosquito DNA

All mosquito-mealworm mixtures produced visible PCR products after amplification. Mosquito-mealworm mixtures containing 0 % and 100 % *Ae. aegypti* produced readable sequences that provided species-level matches to either *T. molitor* (0 % mosquito) or *Ae. aegypti* (100 % mosquito) (Table 3). For each of the other mosquito-mealworm mixes (i.e., 5 %, 10 %, 15 %, 20 % and 25 % mosquito), direct sequencing (without cloning) provided mixed DNA sequences that could not be interpreted (i.e., erroneous sequences) (Table 3). Sequences from clone libraries of each of the different mosquito-mealworm mixtures revealed the presence of both *Ae. aegypti* and *T. molitor*, though the ratio of sequences belonging to *Ae. aegypti* and *T. molitor* from each clone library did not appear to be related to the ratios of mosquito-mealworm in the various mixtures (Table 3). These results indicate that while mosquitoes can be detected when present in low concentrations relative to other prey (*T. molitor*), the proportion of sequences corresponding to mosquito DNA in clone libraries do not correspond to ratios of mosquito-mealworm in the original samples (artificial bat feces).

**Table 3:** Detectability of mosquito DNA in artificial bat feces with increasing concentrations of *Aedes aegypti* (by volume; 0-100 %).

**% *Aedes aegypti* (by volume)**	**0**	**5**	**10**	**15**	**20**	**25**	**100**
Replicate 1	✓	✓✗	✓✗	✓✗	✓✗	✓✗	✗
Replicate 2	✓	✓✗	✓✗	✓✗	✓✗	°	✗
Replicate 3	✓	✓✗	°	✓✗	✓✗	✓✗	✗
% of mosquito sequences in clone library	n/a	20	30	10	30	30	n/a

✗ represents successful PCR amplification with a corresponding DNA sequence matching *Ae. aegypti*; ✓ represents successful PCR amplification with a corresponding DNA sequence matching *T. molitor*; ✓✗ represents mixed DNA sequence; ° represents a non-readable sequence due to excessive loss of PCR products during purification prior to sequencing.

#### Diets of wild trapped bats

**Figure 4:**
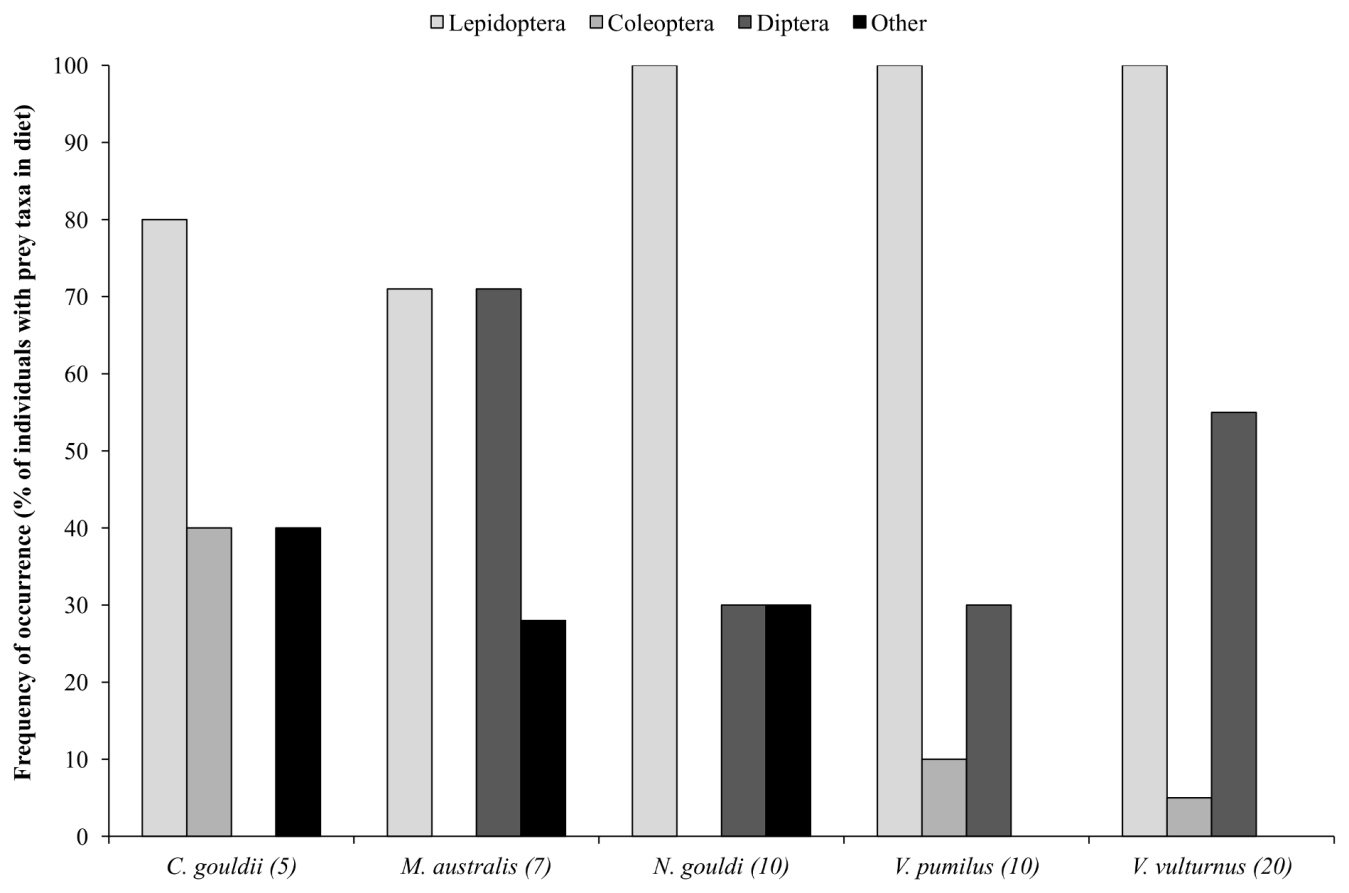
Bat diets. Frequency of occurrence of each insect taxa in the diets of the five insectivorous bats (i.e., percentage of individuals of a species that consumed each insect taxa).

A total of 40 prey taxa were identified from the feces of 52 insectivorous bats representing five species. Lepidopterans were the most frequently detected prey, present in the feces of 49 individuals (Table 4, Figure 4). Coleopterans were only detected in the feces of *C. gouldii*, *V. pumilus* and *V. vulturnus*, but were not commonly present in fecal samples (2 of 5 individuals, 1 of 10 and 1 of 20) (Table 4, Figure 4). Dipterans and ‘other’ taxa were detected in the feces of four species (Table 4, Figure 4). *Aedes vigilax* was detected only in the feces of two bat species (*V. pumilus* and *V. vulturnus*). Mosquitoes were present in the feces of 2 of 10 *V. pumilus* individuals, both of which were trapped during neap tides (Jan and Mar). Mosquitoes were detected in the feces of 11 of 20 *V. vulturnus* individuals, trapped during spring and neap tides (Dec-Feb). 

**Table 4:** Nearest matches and percentage similarity of DNA sequences obtained from the feces of each insectivorous bat species.

Order	Family	Genus	Species	% similarity to nearest match on BOLD
***C. gouldii***				
Blattodea	Blaberidae	*Geoscapheus*	Unknown sp.	96.08
Coleoptera	Unknown	Unknown	Unknown sp.	99.36
Lepidoptera	Geometridae^+^	*Dysbatus*	*singularis*	100.00
	Geometridae^+^	*Nisista*	Unknown sp.	98.72
	Xyloryctidae	*Cryptophasa*	Unknown sp.	98.33
***M. australis***				
Blattodea	Unknown	Unknown	Unknown sp.	94.23
Diptera	Drosophilidae	*Drosophila*	Unknown sp.	98.04
	Hippoboscidae	Unknown	Unknown sp.	98.72
Lepidoptera	Oecophoridae	*Antipterna*	*tricella*	99.35
	Geometridae^+^	*Dysbatus*	*singularis*	100.00
***N. gouldi***				
Blattodea	Unknown	Unknown	Unknown sp.	94.23
Diptera	Drosophilidae	*Drosophila*	Unknown sp.	98.04
Hemiptera	Cicadidae	*Psaltoda*	*plaga*	100.00
Lepidoptera	Crambidae^+^	*Orthospila*	Unknown sp.	97.44
	Noctuidae^+^	*Lysimelia*	*lenis*	100
***V. pumilus***				
Coleoptera	Unknown	Unknown	Unknown sp.	97.83
Diptera	Tabanidae	Unknown	Unknown sp.	100.00
	Culicidae	*Aedes*	*vigilax*	100.00
		*Aedes*	Unknown sp.	97.44
Lepidoptera	Geometridae^+^	*Scioglyptis*	*lyciaria*	100.00
	Limacodidae	*Pseudanapaea*	*denotata*PS1	100.00
	Noctuidae^+^	*Mythimna*	*convecta*	100.00
	Oecophoridae	*Antipterna*	*tricella*	99.35
	Pyralidae^+^	*Spectrotrota*	*fimbrialis*	100.00
	Xyloryctidae	*Thymiatris*	Unknown sp.	97.37
***V. vulturnus***				
Coleoptera	Unknown	Unknown	Unknown	98.08
Diptera	Culicidae	*Aedes*	*vigilax*	100.00
		*Aedes*	Unknown sp.	98.69
Lepidoptera	Choreutidae	*Brenthia*	Unknown sp.	98.69
	Cosmopterigidae	*Limnaecia*	sp. GC14	99.34
	Crambidae^+^	*Maruca*	Unknown sp.	97.56
		*Eurrhyparodes*	*bracteolalis*	99.31
	Geometridae^+^	*Nearcha*	Unknown sp.	98.69
	Noctuidae^+^	*Achaea*	Unknown sp.	97.73
		*Characoma*	*vallata*	99.35
		*Ericeia*	Unknown sp.	97.28
	Nymphalidae	*Acraea*	*andromacha*	99.35
	Oecophoridae	*Barea*	Unknown sp.	98.08
		*Oligoloba*	Unknown sp.	98.69
	Unknown	Unknown	Unknown sp.	100.00

'Unknown' labels are provided if percent similarity to nearest match was not sufficient to assign the match to a particular taxa, or if reference sequences were not designated a taxon label. ^+^ Represents lepidopteran families with tympanal organs. See Supporting Information – Results S1 for DNA sequences.

*Chalinolobus gouldii*, *N. gouldi*, *V. pumilus* and *V. vulturnus* all consumed prey in proportion to the abundance of arthropods in light trap collections (χ^2^=0.324, P=0.955; χ^2^=2.773, P=0.428; χ^2^=1.637, P=0.651; χ^2^=1.637, P=0.651).

Correlation analyses revealed no significant relationships between *Ae*. *vigilax* consumption and abundance of lepidopterans, coleopterans, other insects or all insects combined (r(7)=0.018, P=0.969; r(7)=0.274, P=0.552; r(7)=-0.226, P=0.627; r(7)=0.178, P=0.703). A significant positive relationship between *Ae*. *vigilax* consumption and dipteran (culicid and non-culicid) abundance was observed (r(7)=0.889, P=0.007). 

## Discussion

This study used prey DNA within bat feces to investigate whether consumption of mosquitoes was restricted to small bats with high frequency echolocation calls in the study area, whose activity was correlated with mosquito abundance [17]. Although the diets of all bat species reflected the abundance of prey taxa in light trap collections, mosquitoes were only detected in the feces of the two smallest bats (*V. pumilus* and *V. vulturnus*). However, the proportion of individuals of these species that consumed mosquitoes was low. Although mosquitoes were not a common prey item consumed by smaller sized bats in coastal forest, it is not possible to fully assess their importance as a prey item for bats in the study area as bats were not trapped in saltmarsh where small prey might be most efficiently preyed upon.

### Limitations of dietary study

Since sampling of bats was undertaken in the course of one field season, the sample size for most species investigated was relatively small. Additionally, some DNA sequences had low (<98 %) similarity to reference sequences in BOLD and these identifications should be treated conservatively. Consequently, interpretation of dietary results must be taken with caution. Although it was possible to identify prey in bat feces using molecular techniques, it remains impossible to quantify the amount of prey consumed. While at least two studies have discussed the potential use of clone library proportions to infer quantitative information about consumed prey [37,38], given the variable percentage of prey sequences identified from clone libraries developed for artificial bat feces in this study as well as the variability associated with DNA degradation rates of different prey taxa [39], this information is likely to be unreliable and ambiguous. The molecular technique provided high resolution about consumed prey, however, the low number of identified prey for each individual bat (1-3 prey/bat) is only likely to represent a subset of all prey consumed by an individual bat. While this limitation was avoided in a previous study [40] by separating insect fragments from within bat feces prior to the application of molecular techniques (e.g., PCR), it is possible that many soft-bodied prey without chitinous body parts may be overlooked using this technique. The use of next-generation sequencing applications (e.g., pyrosequencing) in studies of bat diet (e.g., [41]) may also allow for the detection of more taxa than standard DNA techniques (cloning and Sanger sequencing). However, given the variable degradation of DNA of different taxa as well as the variability associated with mtDNA copy-numbers, it is likely that quantification of consumed prey will remain limited.

### Prey abundance

While fourteen mosquito species were represented in CO_2_-baited EVS traps during the study, *Ae. vigilax* was consistently the most abundant, irrespective of tidal cycle. This trend has been observed during long-term mosquito surveillance in the study area, in which *Ae. vigilax* represented 41.2 % of all mosquitoes trapped over nine consecutive trapping seasons (unpublished data – L. Gonsalves and C. Webb). The consistent presence of highly abundant populations of this species in the study area provides bats, particularly small sized species, with a consistent prey resource during summer. Nightly abundance of *Aedes vigilax* populations recorded in the forest habitat during this study (364.67 ± 86.96 and 229.10 ± 60.00 during neap and spring tides) was comparable to nightly abundance of *Ae*. *vigilax* populations recorded in saltmarsh habitats over the same period in 2008-09 (528.2 ± 347.9 and 261.1 ± 127.5 during neap and spring tides) [17].

The most abundant taxa in light trap collections were lepidopterans. While it is acknowledged that certain insect taxa may be more attracted to particular attractant traps [42] and therefore the relative abundance of these taxa can be overestimated, light trapping is commonly used to measure insect abundance and can be used to investigate temporal trends in local insect abundances [43]. Coleopterans, dipterans and ‘other’ insects were also present in light trap collections, but were significantly less abundant in traps than lepidopterans. Similar trends in insect abundances have been observed in other habitats (coastal swamp forest) within the study area, with lepidopterans representing the greatest biomass in light traps [18].

Since two different trapping techniques were used to survey mosquito populations and aerial insect fauna, it is not possible to directly compare the abundance of mosquitoes to the abundance of insects in light trap collections. However, the abundance of *Ae*. *vigilax* (229±60 during spring tides and 365±87 during neap tides) suggests that mosquitoes, like lepidopterans, represent a highly abundant prey resource in the study area. 

### Relationships between bat size, diet and mosquito consumption

The diets of the five insectivorous bat species we investigated consisted of a diverse range of prey. Previous dietary studies of the five bat species also report a diverse range of prey [44,45,46]. We found that lepidopterans were the most frequently detected insects in the feces of all bat species, ranging from 71 to 100 % frequency of occurrence with only six of 52 bats not having lepidopteran DNA in their feces. Lepidopterans were also the most abundant insect taxa in light trap collections at each site. We assume that the bats investigated during this study were foraging within the habitats in which they were captured, as echolocation calls of all trapped bat species have previously been detected in the same study sites [17], with three of the five species producing feeding buzzes. 

The prey detected in the feces of all bats reflected the locally abundant prey taxa. However, *Ae. vigilax* was only detected in the feces of *V. pumilus* and *V. vulturnus*. Mosquitoes were detected in < 60 % of individuals of these species (20 % and 55%, respectively), though the numbers consumed are unknown given the molecular techniques used can only provide presence/absence data. These two congeneric species are morphologically similar with echolocation calls that overlap to such a degree that it is not possible to differentiate between the two species in the study area [33]. Both bats are small in size (4 and 4.5 g) and employ high frequency echolocation (51-55 kHz). The absence of mosquitoes from the diets of larger bats suggests that there may be a negative relationship between bat size and consumption of *Ae*. *vigilax*. However, similar to findings from studies of diets of many medium-large sized European bats [24,25,47], two of the medium sized bats in our study (*N. gouldi* and *M. australis*) on occasion consumed prey that were smaller than *Ae*. *vigilax* (*Drosophila* sp., 2-4 mm).

Generally, smaller predators acquire small prey, while larger predators are capable of consuming both, small and large prey [48]. However, this generalisation may not be appropriate for echolocating aerial foraging bats restricted to prey of a certain size due to detectability constraints imposed by echolocation call structure [22]. It is thought that bats that employ high-frequency echolocation (with corresponding short wavelengths) are more suited to detecting small prey [22] such as mosquitoes (<5 mm), than bats that use low-frequency echolocation. Given echolocation call frequencies are negatively associated with bat size [23], any reduction in the ability of larger bats to detect small prey may result in their absence in the diets of these bats. In our study, the presence of small prey in the feces of all high-frequency echolocating bats (and not the low-frequency echolocating *C. gouldii*; 25-34 kHz) supports the suggestions of Barclay and Brigham [22]. 

While *N. gouldi* and *M. australis* both consumed prey smaller than mosquitoes, the absence of mosquitoes from their feces, given mosquito DNA was still detectable when mosquitoes were present as 5 % of insect material in artificial bat feces (approximately equivalent to the mass of 5 mosquitoes), suggests that it is unlikely that larger (> 6 g) bats actively seek mosquitoes as prey in forest. Additionally, *N. gouldi* employs a gleaning foraging strategy, with microscopy revealing a diet predominantly consisting of moths in other areas [46]. Gleaning bats tend to use prey-generated sounds as cues for detection of prey on substrates. It is acknowledged, however, that the level of sensitivity of the molecular technique for the detection of mosquito DNA in artificial feces may be an over-estimate of the detectability of mosquito DNA in the feces of wild caught bats, since no attempts were made to incorporate the effects enzymatic degradation of prey known to occur in the guts of bat species [49]. Additionally, the complexity and diversity of prey in the feces of wild bats is greater than what was used in the artificial feces in this study. 

### How many mosquitoes would Vespadelus need to consume to satisfy energy requirements?

Using the equation of Speakman and Thomas [26], the minimum energy required to maintain day-to-day activity (field metabolic rate) for the two bats species found to consume mosquitoes is 17.89 kJd^-1^ (*V. vulturnus*) and 19.51 kJd^-1^ (*V. pumilus*). If it is assumed that the two bat species were specialist foragers and consumed only mosquitoes, *V. vulturnus* and *V. pumilus* would be required to consume ~604 and ~659 mosquitoes, respectively, each night just to maintain day-to-day activity (assuming a mosquito weighs 0.002 g and provides 14.8 kJg^-1^of energy – [27]). Conversely, if the two bats consumed only lepidopterans of similar size, *V. vulturnus* and *V. pumilus* would need to consume ~164 and ~179 moths, respectively (assuming a moth weighs 0.004 g and provides 27.2 kJg^-1^ of energy - [28]). While this does not consider the relative digestibility, rate of capture, and handling time for mosquitoes and moths, the foraging time required to meet FMR requirements under these two scenarios is likely to be relatively greater for these two species if they selected only mosquitoes as prey. The disparity in foraging time required to meet FMR requirements under these scenarios increases with the mass of bats. Consequently, the profitability of mosquitoes as prey items would be especially low for larger bats with greater energetic demands. This may provide an explanation for the low level of mosquito consumption among the two smallest bat species and the absence of mosquitoes in the feces of larger bats (Table 1).

Despite the low energetic profitability of mosquitoes relative to other prey, bats may still pursue them if they are extremely abundant [50], particularly in open habitats. In this study, the abundance of mosquitoes was comparable to or greater than (during neap tides) the abundance of all insect taxa combined. Consumption of mosquitoes may be more common for smaller bat species when foraging within the less-cluttered saltmarsh habitat in the study area where *Ae*. *vigilax* emerges and proportional feeding activity by bats is greatest [17]. Bats are known to switch habitats to select alternative, more abundant prey despite no apparent shortage of profitable prey [50]. Similarly, in our study area, bats have been found to shift habitat use from larval to refuge habitats of *Ae*. *vigilax* in association with fluctuations in the abundance of the mosquito in the two habitats [18]. 

### Implications for broad-scale mosquito control

While it is beyond the scope of this study to infer potential impacts of broadscale mosquito control and the associated reductions in larval mosquito populations on insectivorous bat diet and health, declines in bat populations have previously been attributed to deteriorating feeding conditions [51]. In Australia, although broadscale control of mosquito populations is generally only undertaken during periods of epidemic disease activity, early season treatment to suppress irruptions of mosquito populations later in the season is becoming increasingly common [4]. Mosquito control activities designed to reduce the abundance of mosquitoes, and not complete eradication, may diminish larval mosquito populations by as much as 98.2 % [4], substantially reducing the availability of mosquitoes to foraging bats. Although mosquitoes were only preyed on by two smaller species of bats, they were consumed by > 50 % of individuals for *V. vulturnus*. This species is likely to be most sensitive to reductions in mosquito abundance and should be monitored during mosquito control activities. 

## Supporting Information

Methods S1 (DOCX)

Results S1 (DOCX)
