# Supplementary material for: Mosquito Consumption by Insectivorous Bats: Does Size Matter?
Source: PLoS One. 2013 Oct 10;8(10):e77183. doi: 10.1371/journal.pone.0077183 (PMC3795000; doi:10.1371/journal.pone.0077183)
Supplement: Methods S1 — (DOCX) [file pone.0077183.s001.docx]

**Supporting Information – Methods S1**

*Analysis of faecal samples*

Genomic DNA was extracted from a pooled sample of five faecal pellets for each trapped individual using the Ultraclean Fecal DNA kit (Mo Bio Laboratories) for *V. vulturnus* or the QIAamp DNA Stool Mini Kit (Qiagen) for all other bats. The Ultraclean Fecal DNA kit was used according to manufacturer’s instructions, after the faecal pellets were broken apart with sterile toothpicks. The QIAamp stool mini kit was used to extract DNA as described by Zeale *et al*. [[1](#_ENREF_1)].

PCRs reactions were based on the Kappa Robust HS PCR kit and consisted of Buffer A (5X concentrate), forward and reverse primers (5 μM), MgCl_2_ (25 mM), dNTPs mix (10 mM), Kapa2G Robust HS polymerase (0.5 U) and made up to 10 μL reactions using sterile milliQ water. All PCRs were carried out using an Eppendorf EG thermocycler.

Taxon-specific primers, ZBJ-ArtF1c and ZBJ-ArtR2c [[1](#_ENREF_1)] were used to amplify a 157 bp section of the DNA barcoding region, cytochrome oxidase I with the following cycling conditions: initial denaturation at 95° C for 3 minutes, followed by 16 cycles of 95° C for 30 s, 61-53° C (-0.5° C per cycle) for 30 s, 72° C for 30 s, followed by an additional 24 cycles of 95° C for 30 s, 53° C for 30 s, 72° C for 30 s and a final extension of 72° C for 10 m. Gel electrophoresis (1 % agarose) was used to verify the presence of a PCR product of the correct size.

Samples that failed to produce visible PCR products were subjected to a nested PCR, in which samples were initially amplified using a universal mini-barcoding primer, Uni-MinibarF1 [[2](#_ENREF_2)] and ZBJ-ArtR2c. The cycling conditions consisted of an initial denaturation at 95° C for 3 m, followed by 5 cycles of 95° C for 30 s, 46° C for 30 s and 72° C for 30 s, and a further 35 cycles of 95° C for 30 s, 53° C for 30 s, 72° C for 30 s and a final extension of 72° C for 5 m. One microlitre of PCR product was then used as the template in a second round of amplification using taxon-specific primers.

Duplicate PCR products generated using the taxon-specific primers were pooled, purified using Ultraclean PCR clean-up kit (Mo-Bio laboratories) and cloned using either a pGem-T-easy vector cloning kit and JM109 high competency *E. coli* cells (Promega), or an Acquire PCR Cloning Kit and high efficiency competent cells (Alchemy Biosciences) according to manufacturers’ instructions. A selection of clones were lysed in 50 μL of Sterile MilliQ water at 95° C and 1 μL was subjected to PCR to amplify DNA inserts, using vector primers T7 and SP6 (10 μM), and GoTaq green mastermix (Promega). Gel electrophoresis was used to verify insert size. Sixteen clones from each bat, with an insert of appropriate size (ca.320 bp) were purified using the Ultraclean PCR purification kit (Mo Bio Laboratories) and sequenced at the Australian Genome Research Facility (Westmead Millennium Institute, Sydney) using the ABI Prism Big Dye Terminator reaction on a AB3730xl sequencer. DNA sequences were aligned using Clustal X [[3](#_ENREF_3)] and trimmed of flanking vector bases in BioEdit 7.0.0 [[4](#_ENREF_4)]. All spurious sequences (those shorter than ca.157 bp) were excluded from further analysis. All other sequences were entered into the identification engine on the barcoding of life database (BOLD). The nearest sequence match and percent similarity of each sequence was recorded, with a taxonomic assignment to order, family, genus or species using taxonomic assignment thresholds [[1](#_ENREF_1)]. When sequence similarity was greater for a taxon not currently known to exist in Australia, the sequence was assigned to the nearest match known to occur in Australia.

*Calibration of technique sensitivity for detection of mosquito DNA*

While Zeale *et al*. [[1](#_ENREF_1)] reported no bias in detectability of various taxa, the tested taxa did not include mosquitoes. To provide a baseline for detectability of mosquito DNA among DNA of other taxa, artificial bat faecal material was manufactured with mealworms and varying concentrations of mosquitoes (by volume). One batch of each prey (mosquito plus mealworm) was made by grinding known numbers of mosquitoes and mealworms in separate 2-mL vials with distilled water. For the mosquito stock mixture, 0.43 g of *Aedes aegyptii* individuals was added to 2 mL of distilled water (0.18 g mL^-1^ assuming 1 g of mosquito = 1 mL), while 0.44 g of mealworm individuals was added to 1 mL of distilled water for the stock mealworm mixture (0.30 g mL^-1^ assuming 1 g of mealworm = 1 mL). Five sterile glass balls were added before vigorous shaking in a mosavex machine (Department of Medical Entomology, Westmead Hospital and University of Sydney) for 1 h 15 m. Mealworm and mosquito slurries were then transferred to separate sterile jars using 1-mL pipettes. Using the two stock slurries, 0 %, 5 %, 10 %, 15 %, 20 % and 100 % of mosquito solutions were made.

1. Zeale MRK, Butlin RK, Barker GLA, Lees DC, Jones G (2011) Taxon-specific PCR for DNA barcoding arthropod prey in bat faeces. Mol Ecol Resour 11: 236-244.

2. Meusnier I, Singer G, Landry J-F, Hickey D, Hebert P, et al. (2008) A universal DNA mini-barcode for biodiversity analysis. BMC Genomics 9: 214.

3. Larkin MA, Blackshields G, Brown NP, Chenna R, McGettigan PA, et al. (2007) Clustal W and Clustal X version 2.0. Bioinformatics 23: 2947-2948.

4. Hall T (2004) BioEdit. Biological sequence alignment editor for Win95.
