# Supplementary material for: Mosquito Consumption by Insectivorous Bats: Does Size Matter?
Source: PLoS One. 2013 Oct 10;8(10):e77183. doi: 10.1371/journal.pone.0077183 (PMC3795000; doi:10.1371/journal.pone.0077183)
Supplement: Results S1 — (DOCX) [file pone.0077183.s002.docx]

**Supporting Information – Results S1**

*Chalinolobus gouldii*

>CG1_Geoscapheus

AGCATGAGCAGGTATAGTTGGAACCTCCTTAAGTATATTAATTCGAGCAGAATAAAATCAACCTGCATCCTTAATTGCTGACGATCAAATTTATAATGTAATTGTTACAGCTCACGCTTTAATTATAAATTTTTTAATAGTTATGCCAATTTTGAAT

>CG2_Coleoptera

TGCTTGATCAGGAATAGTAGGAACTTCATTAAGTATACTAATTCGAGCTGAATTAGGAAATCCTGGCTCCTTAATTGGAGATGATCAAATTTATAATGTTATTGTAACAGCTCACGCTTTTGTAATAATTTTCTTCATAGCAATGCCTATTATGATT

>CG3_Dysabatus_singularis

TATTTGAGCGGGGATAGTTGGAACATCTTTAAGATTACTAATTCGAGCAGAATTGGGTAACCCAGGATCTTTAATCGGAGATGATCAAATTTATAATACTATTGTAACTGCACATGCTTTTATTATAATTTTTTTTATAGTTATACCAATTATAATT

>CG_4_Nisista

TATTTGAGCAGGAATAGTGGGAACATCATTAAGATTATTAATTCGAGCAGAATTAGGTAATCCAGGATCTTTAATTGGAGATGATCAGATTTATAATACTATTGTAACTGCACATGCTTTTATTATAATTTTTTTCATAGTTATAGCAATTTTAATT

>CG_5_Cryptophasa

AATTTCATCAGGAATAGTTGCAACTTCCTTAAGTTTACTTATTCGAGCTGAATTAGGAAACCGCGGATCTTTAATCGGAGATGATCAAATTTATAATACAATCGTAACAGCTCATGCTTTTATTATAATTTTCTTTATACTTATACCTATTATAAT

*Miniopterus australis*

>MA1_Blattodea

TGCATGATCAGGGATAGTAGGTACCTCATTAAGTATATTAATTCGTGCCGAATTAAACCAACCCGGTTCCCTAATTGGGGATGATCAAATCTATAATGTGATTGTTACAGCTCATGCCTTCATTATAATTTTCTTTATAGTAATACCTATTCTCATT

>MA2_Drosophila

AGCTTGAGCTGGAATAGTTGGAACATCTTTAAGAATTTTAATTCGAGCTGAATTACGACATCCTGGAGCATTAATTGCAGATGATCAAATTTATAATGTAATTGTAACTGCACATGCTTTTATTATAATTTTATTTATAGTTATACCTATTATAAAT

>MA3_Hippoboscidae

TATATGATCAGGAATAATTGGAACATCATTAAGTATTTTAATTCGAATAGAATTAGGACATCCAGGAGCATTAATTGGTAATGATCAAATTTATAATATAATTGTAACTTCCCATGCATTTATTATAATTTTTTTCATAGTAATTCCAATTATAATT

>MA4_Antipterna_tricella

AATTTGGGCAGGAATAGTTGGAACATCATTAAGACTACTTATTCGAGCTGAATAGGAAACCCTGGATCATTAATTGGTGATGACCAAATTTATAATACTATTGTTACTGCTCATGCCTTTATTATAATTTTTTTATAGTAATACCAATCATAATT

>MA5_Dysabatus_singularis

TATTTGAGCGGGGATAGTTGGAACATCTTTAAGATTACTAATTCGAGCAGAATTGGGTAACCCAGGATCTTTAATCGGAGATGATCAAATTTATAATACTATTGTAACTGCACATGCTTTTATTATAATTTTTTTTATAGTTATACCAAATATAAAT

*Nyctophilus gouldi*

>NG1_Blattodea

TGCATGATCAGGGATAGTAGGTACCTCATTAAGTATATTAATTCGTGCCGAATTAAACCAACCCGGTTCCCTAATTGGGGATGATCAAATCTATAATGTGATTGTTACAGCTCATGCCTTCATTATAATTTTCTTTATAGTAATACCTATTCTCATT

>NG2_Drosophila

AGCTTGAGCTGGAATAGTTGGAACATCTTTAAGAATTTTAATTCGAGCTGAATTACGACATCCTGGAGCATTAATTGCAGATGATCAAATTTATAATGTAATTGTAACTGCACATGCTTTTATTATAATTTTATTTATAGTTATACCTATTATAAAT

>NG3_Psaltoda_plaga

TATTTGATCAGGAATAGTGGGGACTGCTTTGAGAGTTTTAATTCGAGTTGAATTAGGAACCCCGGGTTCTTTTATTGGTGATGACCAAATTTACAATGTAATTGTAACAGCACATGCATTTATTATAATTTTTTTTATAGTTATACCAATTATAATT

>NG4_Orthospila

AATTTGAGCTGGAATAGTAGGAACATCTTTAAGACTATTAATTCGAGCAGAATTAGGAAATCCAGGTTCTCTTATTGGAGATGATCAAATTTATAATACTATTGTAACTGCTCATGCTTTTATTATAATTTTTTTTATAGAATTACCGATTTTAATT

*Vespadelus pumilus*

>VP1_Coleoptera

TGCTTGATCAGGAATAGTAGGAACTTCATTAAGTATAATAATTCGAGCTGAATTAGGAAATCCTCGCTCCTTAATTGGAGATGATCAAATTTATAATGTTATTGTAACAGCTCACGCATTTGTAATAATTTTCTTCATAGCAATGCATATTATGAAT

>VP2_Tabanidae

GGCATGAGCTGGAATAATTGGAACATCATTAAGAATTTTAATTCGAGCAGAATTAGGACACCCGGGAGCTTTAATTGGTGATGATCAAATCTACAATGTTATTGTAACAGCACATGCTTTTGTCATAATTTTCTTTATAGTAATACCTATTATGATT

>VP3_Aedes_vigilax

AGTTTGATCTGGAATAGTAGGAACATCATTAAGAGTACTAATTCGTGCTGAATTAAGTCATCCAGGTATATTTATTGGAAATGATCAAATTTATAATGTAATTGTTACAGCTCATGCATTTATTATAATTTTCTTTATAGTTATACCTATTATAATT

>VP4_Aedes

AGTTTGATCTGGAATAGTAGCAACATCATTAAGAGTACTAATTCGTGCTGAATTAAGTCATCCAGGTATATTTATTGGAAATGATCAAATTTATAATGTAATTCTTACAcCTCATGCATTTATTATAAATTTCTTTATAGTTATACCTATTATAATT

>VP5_Scioglyptis_lyciaria

AATTTGAGCTGGAATAGTTGGAACTTCATTAAGATTATTAATTCGAGCAGAATTAGGTAATCCTGGATCCTTAATTGGTGATGACCAAATTTATAATACTATTGTAACTGCTCATGCTTTTATTATAATTTTCTTTATAGTTATACCAAATATAAAT

>VP6_Pseudanapaea_denotata

AATTTGATCAGGAATAGTGGGTACTTCTCTTAGATTACTTATTCGAGCTGAATTAGGTAATCCAGGATCATTAATTGGAGATGATCAAATTTATAATACTATTGTTACAGCACATGCTTTTATTATAATTTTTTTTATAGTAATACCAATTATAATT

>VP7_Mythimna_convecta

AATTTGAGCTGGTATAGTTGGAACTTCATTAAGATTACTAATTCGAGCTGAATTAGGAACCCCTGGATCTTTAATTGGAGATGATCAAATTTATAATACTATTGTTACAGCTCATGCTTTTATTATAATTTTTTTTATAGTTATACCTATTATAATC

>VP8_Antipterna_tricella

AATTTGGGCAGGAATAGTTGGAACATCATTAAGACTACTTATTCGAGCTGAATTAGGAAACCCTGGATCATTAATTGGTGATGACCAAATTTATAATACTATTGTTACTGCTCATGCCTTTATTATAATTTTTTTTATAGTAATACCAATCATAATT

>VP9_Spectrotrota_fimbrialis

AATTTGATCAGGAATAGTTGGAACTTCTTTAAGTTTATTAATTCGTGCTGAATTAGGGAATCCTGGATCATTAATTGGAGATGATCAAATTTATAATACTATTGTTACTGGACATGCATTTATTATAATTTTTTTTATAGTTATACCTATTATAATT

>VP10_Thymiatris

AATTTGAGCAGGAATCGTAGGAACTTCTTTAAGTCTTCTTATTCGAGCAGAATTAGGAAATCCAGCATCCTTAATTGCAGATGATCAAATTTATAATACAATTGTTACAGCACATGCATTTATTATAAATTTTTTAATAGTAATACCAATTATAAAT

*Vespadelus vulturnus*

>VV1_Coleoptera

TGCTTGATCAGGAATAGTAGGAACTTCATTAAGTATAATAATTCGAGCTGAATTAGGAAATCCTGGCTCCTTAATTGGAGATGATCAAATTTATAATGTTATTGTAACAGCTCACGCTTTTGTAATAATTTTCTTCATAGCAATGCATATTATGATT

>VV2_Aedes_vigilax

AGTTTGATCTGGAATAGTAGGAACATCATTAAGAGTACTAATTCGTGCTGAATTAAGTCATCCAGGTATATTTATTGGAAATGATCAAATTTATAATGTAATTGTTACAGCTCATGCATTTATTATAATTTTCTTTATAGTTATACCTATTATAATT

>VV3_Aedes

AGTTTGATCTGGAATAGTAGGAACATCATTAAGAGTACTAATTCGTGCTGAATTAAGTCATCCAGGTATATTTATTGGAAATGATCAAATTTATAATGTAATTCTTACAGCTCATCCATTTATTATAATTTTCTTTATAGTTATACCTATTATAAAT

>VV4_Brenthia

ATTTTGATCAGGAATAGTTGGAACTTCTCTAAGTTTATTAATTCGAGCTGAATTAGCTAATCCTCGATCATTAATTGGAGATGATCAAATTTATAATACTATTGTGACAGCTCATGCTTTTATCATAATTTTTTTAATAGTGATGCCAATTATAATT

>VV5_Limnaecia_sp.GC14

AATTTGAGCAGGAATAGTAGGACTTCTCTTAGTTTATTAATTCGAGCTGAATTAGGAAATCCAGGATCTTTAATTGGAGATGATCAAATTTATAATACTATTGTAACTGCTCATGCATTTATTATAATTTTTTTTATAGTTGTACCTATTATAATC

>VV6_Maruca

AATTTGAGCAGGAATAGTAGCAACATCTTTAAGTTTATTAATTCGAGCAGAATTAGCTAATCCTGGATCTTTAATTGGAGATGATCAAATTTATAATACTATTGTAACACCTCATGCATTTATTATAAATTTTTTTATAGTAATACCTATTATAAAT

>VV7_Eurrhyparodes_bracteolalis

AATTTGAGCTGGAATAGTAGGGACATCTTTAAGTTTATTAATTCGAGCTGAATTAGGAACCCCTGGATCATTAATTGGAGATGATCAAATTTATAATACTATTGTAACAGCTCATGCATTTATTATAATTTTTTTAATAGTTATGCGATTTATAATT

>VV8_Nearcha

AATTTGAGCTGGAATAATAGGAACTTCATTAAGATTACTAATCCGAGCTGAATTAGGTAATCCTGGCTCTTTAATTGGAGACGACCAAATTTATAATACTATTGTAACTGCTCATGCATTTATTATAATTTTTTTTATAGTTATACCTATTATAAAA

>VV9_Achaea

TATTTGAGCAGGAATAGTAGGAACCTCTTTAAGTTTATTAATTCGAGCTGAACTAGGAAATCCCGGATCATTAATGGGAGATGATCAAATTTATAATACTATTGTAACGGCTCATGCTTTTATAATAATTTTTTAAAAACTTAAAGGAATAATAAAT

>VV10_Characoma_vallata

AATTTGAGCTGGAATAGTAGGTACTTCTTTAAGTCTTCTTATTCGAGCTGAATTAGGTAACCCAGGTTCTTTAATTGGCGATGATCAAATTTATAATACTATTGTAACTGCTCATGCATTTATTATAATTTTTTTTATAGTTATACCAATTATAAAT

>VV11_Ericeia

AATTTGAGCAGGAATAGTAGGAACATCTTTAAGTTTATTAATTGGAGCAGAATTAGGAAATCCAGGATCTTTAATTGGAGATGATCAAATTTATAATACTATTGTAACAGCCCATGCTTTTATTATAATTTTTTAAATAGTTATACCATTCATATTT

>VV12_Acraea_andromacha

TATTTGATCTGGAATAATTGGAACATCTTTAAGTTTATTAATTCGAACAGAATTAGGTAACCCAGGATCCTTAATTGGGGATGATCAAATTTATAATACTATTGTTACAGCCCATGCTTTTATTATAAATTTCTTTATAGTTATACCAATTATAAAT

>VV13_Barea

AATTTGAGCAGGAATAGTAGGAACCTCATTAAGTTTACTTATTCGAGCAGAATTAGGTAATCCAGGATCTTTAATTGGGGATGATCAAATTTATAATACCATTGTAGCAGCCCATGCTTTTATTATAATTTTTTTTATAGTTATGCCTACTATAATT

>VV14_Oligoloba

AATTTGAGCAGGAATAGTAGGAACATCTTTAAGACTCTTAATTCGAGCTGAATTAGGAAACCCAGGTTCTTTAATTGGAGATGATCAAATTTATAATACTATTGTTACAGCTCATGCTTTTATTATAAATTTCTTAATAGTTATACCTATTATAAAT

>VV15_Lepidoptera

AATTTGAGCAGGAATAGTTGGAACATCCCTAAGTTTATTAATTCGAGCAGAATTAGGTAATCCTGGATCTTTAATTGGAGATGATCAAATTTATAATACTATTGTAACAGCTCATGCTTTTATTATAATTTTTTTTATAGTTATACCTATTATAATT
